# Mind wandering state detection during video-based learning via EEG

**DOI:** 10.3389/fnhum.2023.1182319

**Published:** 2023-05-30

**Authors:** Shaohua Tang, Yutong Liang, Zheng Li

**Affiliations:** ^1^School of Systems Science, Beijing Normal University, Beijing, China; ^2^International Academic Center of Complex Systems, Beijing Normal University, Zhuhai, China; ^3^Center for Cognition and Neuroergonomics, State Key Laboratory of Cognitive Neuroscience and Learning, Beijing Normal University, Zhuhai, China

**Keywords:** mind wandering, electroencephalography (EEG), passive brain-computer interfaces (pBCI), distance learning, Riemannian geometry, brain-computer interfaces

## Abstract

The aim of this study is to explore the potential of technology for detecting mind wandering, particularly during video-based distance learning, with the ultimate benefit of improving learning outcomes. To overcome the challenges of previous mind wandering research in ecological validity, sample balance, and dataset size, this study utilized practical electroencephalography (EEG) recording hardware and designed a paradigm consisting of viewing short-duration video lectures under a focused learning condition and a future planning condition. Participants estimated statistics of their attentional state at the end of each video, and we combined this rating scale feedback with self-caught key press responses during video watching to obtain binary labels for classifier training. EEG was recorded using an 8-channel system, and spatial covariance features processed by Riemannian geometry were employed. The results demonstrate that a radial basis function kernel support vector machine classifier, using Riemannian-processed covariance features from delta, theta, alpha, and beta bands, can detect mind wandering with a mean area under the receiver operating characteristic curve (AUC) of 0.876 for within-participant classification and AUC of 0.703 for cross-lecture classification. Furthermore, our results suggest that a short duration of training data is sufficient to train a classifier for online decoding, as cross-lecture classification remained at an average AUC of 0.689 when using 70% of the training set (about 9 min). The findings highlight the potential for practical EEG hardware in detecting mind wandering with high accuracy, which has potential application to improving learning outcomes during video-based distance learning.

## 1. Introduction

A passive brain-computer interface (pBCI) is a type of BCI that does not require the user to actively generate signals or perform tasks to interact with the system. Instead, pBCIs use techniques such as electroencephalography (EEG) to record brain activity from healthy people in real-life situations and are suited for applications such as emotion recognition, stress level measurement, and mental workload measurement (Aricò et al., 2018). In the current study, we are interested in building an EEG-based pBCI system to detect mind wandering during video-based learning.

Mind wandering is usually defined as task-unrelated thoughts or stimulus-independent thoughts (Smallwood and Schooler, 2015; Andrews-Hanna et al., 2018). With the wide-spread usage of personal computers, the increasing availability of open courses, and the impact of the pandemic in recent years, video-based distance learning is becoming a vital learning paradigm for many students. However, the frequency of mind wandering during video-based learning has been found to be higher (Risko et al., 2012, 2013) compared to classroom learning. Researchers believe that mind wandering may occur much more frequently outside the laboratory, because there are more temptations in real-world settings (Szpunar et al., 2013). Since students who report higher mind wandering rates during learning have lower test performance, both in video-based learning (Risko et al., 2012) and classroom learning scenarios (Wammes et al., 2016b), developing methods to detect mind wandering during video-based learning has important potential benefits.

Many studies have tried to detect the mind wandering state, however, they were conducted in laboratory settings with well-controlled stimuli (Jin et al., 2019; Dong et al., 2021; Groot et al., 2021; Chen et al., 2022). Particularly, Chen et al. (2022) designed a multi-modal sustained attention to response task (MM-SART), in which participants were instructed to press a key when non-target stimuli appeared and to refrain from doing so when target stimuli appeared. The mental states of the participants were measured via thought probes administered at the end of each experimental block. Chen et al. (2022) observed that entropy-based features led to high classification performance for mind wandering. Below we focus on studies that aim to detect mind wandering state in near-real-life settings. One example study is by Dhindsa et al. (2019) who conducted an experiment in a live lecture scenario using a 16-channel EEG system. They reported 80%–83% accuracy for within participant 2-class classification. In their paradigm, the mind wandering state was obtained from thought probes.

The thought probe is a widely adopted paradigm. However, it has the disadvantage of being intrusive (Weinstein, 2018). If the goal is to build a classification model for real-time mind wandering detection, additional challenges must be addressed. First, since participants are more likely to mind wander with longer inter-probe interval (Seli et al., 2013), probes cannot be used too often, which limits data sample size. Additionally, the samples collected may suffer from an imbalance of wandering versus not, which can lead to poor classifier performance. For example, Dong et al. (2021) reported an on-task versus mind-wandering sample size balance of approximately 3:4, while Dhindsa et al. (2019) reported a ratio of 32:17.

To meet these challenges, some studies have developed new methods to label samples based on the experimental task. In a study conducted by Liu et al. (2013), 24 participants performed English listening tests under 2 scenarios, one with interference and the other without. The interference consisted of distracting conversations, designed to induce an inattentive state. The actual states experienced by the participants were confirmed via report and via video footage examined offline, and EEG signals were recorded via dry electrode. The authors reported classification accuracy of 76.82% with a support vector machine (SVM) classifier. Kaushik et al. (2022) conducted a study with 24 participants performing Tibetan monastic debate tasks, and the attentional state of the participants was rated by 3 observers of video recordings. The group level decoding performance of attention vs. distraction was high when using a long short-term memory neural network classifier (95.86% accuracy); the authors acknowledged that a limitation of the second-observer method was that only clear instances could be annotated. Zhigalov et al. (2019) conducted a magnetoencephalography study in which participants engaged in mindful meditation, a future planning task, and an anxiety-inducing task; the latter two tasks were used to induce wandering thoughts. The authors found that connectivity- and spectral-based classification approaches had similar accuracy, both around 60%.

Mind wandering is commonly thought to be an automatic diversion of attention from a current task, but people can engage in mind wandering intentionally (voluntarily). Intentional mind wandering occurs frequently in everyday life (McVay et al., 2009). A study conducted in a real classroom environment found that more than half of the mind wandering occurrences were intentional (Wammes et al., 2016a). For the content of mind wandering, Seli et al. (2017) found that intentional mind wandering was more future oriented than unintentional (self-initiated but involuntary) mind wandering. Many factors may be related to mind wandering, and their relationships have been shown to be complicated. For example, participants’ motivational level was more relevant to intentional mind wandering compared to unintentional mind wandering, and they tend to reduce intentional mind wandering under high motivation (Seli et al., 2015, 2016c; Robison et al., 2020). Seli et al. (2016a) reported that participants experienced more intentional mind wandering in easy tasks than in difficult tasks and more unintentional mind wandering in difficult tasks than in easy tasks. Thus these factors should be considered in experimental design.

In the current study, we define mind wandering as thoughts unrelated to video lecture content. A realistic video-lecture-based distance learning scenario was created and an 8-channel gel-based EEG system was used to record brain activity of participants under 2 conditions, focused learning and future planning, with the latter condition designed to increase the probability of mind wandering. An aim of this design was to avoid interrupting the ongoing video watching while obtaining reliable labels for the EEG data. We then applied Riemannian geometry-based feature generation and machine learning methods to classify mind wandering versus focused learning in a within-participant and cross-lecture fashion. The high decoding performance of our classifier shows its feasibility and the real world application potential of our pBCI paradigm.

## 2. Materials and methods

### 2.1. Participants

Fourteen participants were recruited (6 females; average age 23.36 ± 4.75). All participants reported no history of neurological disorders, and had normal or corrected-to-normal vision. The experiment was approved by the Ethics Review Committee of the School of Psychology of Beijing Normal University (Approval number: 20221121118). Written informed consent was obtained from each participant before the experiment. Previous mind wandering classification studies, such as Dhindsa et al. (2019), have used data from 15 participants. The small deviations in decoding performance between participants (Supplementary Tables 1, 2) suggest that the number of participants in our study was adequate.

### 2.2. Data acquisition

An 8-channel gel-based EEG system was used for EEG recording (Yiwu Jielian Electronic Technology Co., Ltd., China). The channel locations were F3, F4, T3, C3, C4, T4, O1, and O2 (International 10–20 EEG system) and referenced to an electrode at Cz. The impedance values of the electrodes were kept lower than 80 kΩ as recommended by the manufacturer. While this is higher than typical laboratory EEG standards, it reflects a practical level of impedance achievable during real world usage. Sampled at 1,000 Hz, the EEG signal was high-pass filtered with a passive, 1st order RC filter at 0.3 Hz and low-pass filtered with a 2nd order Bessel filter at 80 Hz. Experiments were conducted in a sound and electromagnetically shielded room.

### 2.3. Procedure

The lecture videos used in the current study were downloaded from a Chinese domestic massive open online course platform^1^ and included 13 disciplines (Physics, Psychology, Chemistry, Economics, Art, and others). We selected videos with length 5 to 10 min, obtaining 362 videos in total. A short video length was chosen to (1) avoid the total experiment length from becoming too long, and (2) to reduce the possibility of mind wandering during the focused learning condition (Khan, 2012).

Prior to the experiment, each participant was first asked to provide 2 images that were most related to his or her personal future plans and frequently appeared in his or her mind wandering episodes during the previous week. These images were to be used as cues before the future planning condition videos. We provided the titles of all videos as well as a brief introduction to their contents, sorted by subject matter. Then each participant was asked to select 2 videos that most interested him or her (to be used for the focused learning condition) as well as 3 videos that were the most uninteresting (to be used for the future planning condition). Then each participant watched all 3 selected uninteresting videos and ranked them; the 2 most uninteresting videos were used for the future planning condition. Note that this means the participants watched the uninteresting videos twice, once during selection and once during the experiment. This re-watching paradigm (Martin et al., 2018) served a dual purpose in our study. First, it helped confirm that participants were genuinely disinterested in the videos, since the content may have exceeded their expectations based on the title and description. Second, it increased the likelihood of mind wandering during the second viewing, which was crucial for our investigation.

**FIGURE 1:**
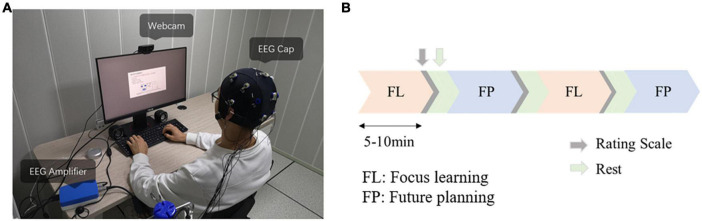
**(A)** Photo of experimental setup. **(B)** Timeline of experiment. Focused learning condition (FL) and future planning condition (FP) alternated, each video lasted 5–10 min, with rating scale feedback and rest between videos. Half of the participants performed FL first and the other half performed FP first.

The experimental environment is shown in Figure 1A. The presentation of all stimuli occurred on an ASUS 23.8” LED monitor that had a spatial resolution of 1,920 × 1,080 pixels; all stimuli were presented on a gray background. All lecture videos had 480 p resolution (235 × 132 cm on screen), and cue images were 500 × 500 pixels (137 × 137 cm on screen). The participants were positioned in front of the computer screen in such a way that their eyes were around 60 cm away from the display.

For the focused learning condition, before video playing, participants were instructed to focus attention on the lecture video and press a key when they lost their attention, then immediately focus attention back on to the video. A brief discussion with the experimenter about the content of the lecture was held before watching, and participants were told that 1 to 2 questions would need to be answered after the video. The interest, discussion, and expectation of questions combined to increase task demand and reinforced motivation to maintain attention (Seli et al., 2019).

For the future planning condition, each participant was asked to perform personal future planning related to the cue images shown on the screen. Cue images were shown only before the lecture and until participants were ready and pressed a button to start the lecture video. During the playing of the lecture videos, participants were instructed to press a key when they found themselves engaged in the videos, then immediately continue to plan. Thus, *key press in both conditions indicated undesired mental state, and marked data to be excluded from classifier training and testing*. The self-caught method was inspired by Braboszcz and Delorme (2011); we use it here due to its simplicity and compatibility with our goal of not disturbing the task.

The EEG data acquisition phase started with a 1-min recording of resting state with eyes open. To average out order effects, half of the participants started with the focused learning condition and the other half started with the future planning condition (Figure 1B). The selected learning videos had average length of 480.68 ± 95.86 s and the selected uninteresting videos had average length of 470.89 ± 82.60 s. The whole experiment lasted about 2 h (from video selection to finish of all the tasks).

In all conditions, EEG signals and facial videos from a webcam placed above the screen were recorded. The participants were told that the experiment process would be recorded by the webcam. They were allowed to adjust the audio volume at the beginning of video playback. Participants could change video playback rate, fast forward, and rewind during video playback via key press on the computer keyboard. We allowed these actions to mimic real-life video-based learning scenarios and to maintain an appropriate level of difficulty.

After each video, participants were asked to provide feedback via rating scales:

1. “What percentage of time were you focused on the video?”
2. “What percentage of time were you intentionally mind wandering?”
3. “What percentage of time were you unintentionally mind wandering?”
4. “What percentage of mind wandering occurrences were marked with key presses?” (for focused learning); “What percentage of video engagement occurrences were marked with key presses?” (for future planning).
5. “For how long before each key press were you mind wandering?” (for focused learning); “For how long before each key press were you engaged in the video?” (for future planning).

For the scale ranges, the first four questions’ ratings were integers from 1 to 10, which represented 10 uniform intervals from 0 to 100% (e.g., 1 represents <10%, 2 represents 10–20%, 3 represents 20–30%, and so on). A time range in seconds was estimated for the 5th question. Before the experiment, the wording of the rating scales was clarified to the participants (for example, that “mind wandering” represents thoughts that are unrelated to the lecture videos). While the responses to these subjective rating scales were likely noisy, they still provide some useful information for data quality estimation (question 1–4) and data cleaning (question 5). We did not assess the quality of learning after the task, because the video lectures were diverse in topic and did not have associated testing material that was comparable across lectures. Thus, we did not measure the detrimental effect of mind wandering on learning in this study.

### 2.4. EEG data pre-processing

EEGLab (V2021.0) and MATLAB (2020.a, MathWorks Inc., Natick, MA, USA) were used for EEG signal processing. The raw EEG signals were high-pass filtered at 1 Hz and down-sampled to 256 Hz. A notch filter at 48–52 Hz was applied to remove power line noise.

The Artefact Subspace Reconstruction (ASR) plugin of EEGLab was used for de-noising. The resting state data (eyes open) recorded at the beginning of every experiment were used as the reference signal input for the ASR algorithm. All participants were included in the analysis.

### 2.5. Feature extraction

#### 2.5.1. Signal filtering

Electroencephalography frequency bands delta (1–4 Hz), theta (4–8 Hz), alpha (8–13 Hz), and beta (13–30 Hz) were obtained by band-pass filtering the EEG signal using windowed-sinc finite impulse response filters (pop_eegfiltnew function of EEGlab).

#### 2.5.2. Signal segmenting and labeling

**FIGURE 2:**
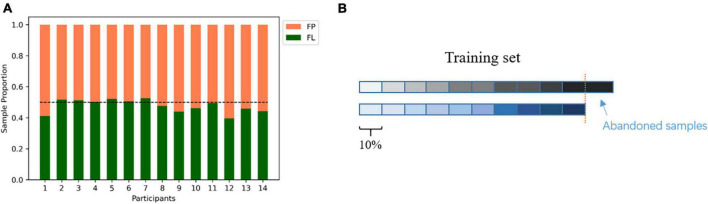
**(A)** Total sample size comparison between future planning (FP, orange) condition and focused learning (FL, green) condition after data cleaning for each participant. Horizontal dotted line represents balanced level (0.5:0.5). Overall, sample sizes were fairly balanced. **(B)** An illustration of the selection of a proportion of the training set, according to temporal order, to test the effect of sample size on classification performance. The two rows of color blocks indicate the EEG samples from 1 FP video and 1 FL video that were used as the training set for cross-lecture prediction. The samples pointed to by the blue arrow were abandoned to keep the training set balanced.

The continuous EEG data were then sliced into 2 s non-overlapping segments. The 2-s window was based on a trade-off between better feature extraction with longer windows versus the need for real-time decoding with shorter windows. The choice of a 2-s window duration is consistent with the approach taken by Kaushik et al. (2022). The segments from the focused learning condition were labeled as non-mind wandering and segments from the future planning condition were labeled mind wandering (2-class). Participants reported occurrences of out-of-task mental state during each condition (that is, mind wandering in the focused learning condition and focus on lecture in the future planning condition) through key press, so based on the key press timings and the duration range provided in the rating scales (we used the higher value of the range given by each participant for a video), any segments which overlapped any key press were excluded in the following analysis. Specifically, let *m* be the high value of the range and *t* be the time of a key press; any data segment which overlapped with [*t*-*m*, *t*] was excluded. Participants had 468 ± 60 (mean ± std) samples in the focused learning condition and 424 ± 50 samples for the future planning condition. The sample size comparison for each participant is shown in Figure 2A.

#### 2.5.3. Riemannian feature extraction

The Riemannian geometry-based approach has achieved state-of-the-art results on various EEG-based BCI, for example, for BCI based on motor imagery (Zanini et al., 2018; Chu et al., 2020), P300 (Li et al., 2020), and SSVEP (Chevallier et al., 2020). It has shown superiority in many related applications, such as for respiratory state classification (Navarro-Sune et al., 2017), EEG artifact detection (Saifutdinova et al., 2019), and decoding of the directional focus of attention (Geirnaert et al., 2021). The approach processes the covariance matrices estimated from EEG segments in their native space, a Riemannian manifold. This manifold can be conceptualized as a “curved space” where calculating the distance between two points (covariance matrices) and calculating the mean of points (mean of covariance matrices) requires a different approach than in Euclidean space (Lotte et al., 2018). The Riemannian approach offers several advantages, including (1) Riemannian manipulations performed in the sensor space are equivalent to those performed in source space (assuming equal dimensionality), which means source localization is not needed (unless the localization process can add information from other aspects), (2) Robustness of the Riemannian mean to outliers, (3) good cross-participant and cross-session generalization ability (Congedo et al., 2017). These characteristics make it a potential tool for pBCI systems. For the conventional usage of the Riemannian approach for EEG feature extraction, one key step is to calculate the geometric mean *P*_𝔊_ of covariance matrices *P*_*i*_, the solution to the following optimization problem [eq. (1)]. In practice, this is solved by an iterative algorithm (Fletcher and Joshi, 2004).


(1) $$P_{𝔊}=𝔊\,\left(P_{1},\ldots ,P_{m}\right)=\underset{P\in P\,\left(n\right)}{\text{argmin}}\sum_{i=1}^{m}\delta_{R}^{2}\,\left(P,P_{i}\right)$$

Here, δ_*R*_ denotes the Riemannian distance. Then, to use standard classifiers, which assume Euclidian space, we must project the data points (covariance matrices) to the Riemann manifold’s tangent space, which captures the local geometry of the manifold at a tangent point by linear approximation. This is performed by eq. (2) and (3) (Barachant et al., 2013):


(2) $$\log_{P}\left(P_{i}\right)=P^{\frac{1}{2}}\,\log\left(P^{-\frac{1}{2}}\,P_{i}\,P^{-\frac{1}{2}}\right)\,P^{\frac{1}{2}}$$

(3) $${\text{s}}_{i}=\mathrm{upper}\,\left(P_{𝔊}^{-\frac{1}{2}}\,\log_{P_{𝔊}}\left(P_{i}\right)\,P_{𝔊}^{-\frac{1}{2}}\right)$$

Here the mean covariance matrix *P*_𝔊_, is the mean calculated from training data. The n by n (number of EEG channels used) covariance matrices are then mapped into *n*(n + 1)/2 dimensional vectors s by the upper(.) operator. The python package “pyRiemann” (Barachant et al., 2022) was used to calculate the above steps.

### 2.6. Classification, performance metric, and statistics

Five kinds of features and 2 classification pipelines were explored in the current study. Riemannian features based on the 4 frequency bands (delta, theta, alpha, and beta) separately as well as their combination were tested. That is, the covariance matrix (across channels) of EEG data from a frequency band was calculated for a data segment (2 s), and the covariance matrix was then processed using the Riemannian approach to obtain a feature vector (sample). For the combination of frequency bands, the vectors from the four bands were concatenated. The two classification pipelines were:

1) Within participant classification. All samples from one participant were used in five-fold cross-validation.
2) Cross-lecture prediction (within participant). Each participant watched 2 different lecture videos for each condition, so there were 4 total videos. Samples from 2 of the 4 videos (one learning, one planning) were used as the training set and samples from the other 2 videos were used as the test set (4 combinations). Here, we further tested the effect of sample size on cross-lecture prediction performance: the datasets corresponding to the two videos used as training were first shortened to the duration of the shorter dataset (to obtain a balanced training set). Then, along the timeline of the video from 10 to 90%, at 10% per step, we took different proportions of samples as the training set, and predict on the entire test set (Figure 2B).

The frequently used machine learning methods in related works (Dong et al., 2021; Chen et al., 2022; Kaushik et al., 2022) were (1) linear support vector machine [SVM(linear)], (2) radial basis function kernel support vector machine [SVM(rbf)], (3) random forest (RF) and (4) logistic regression (LR). We evaluated and compared their classification performance, as measured by the area under the receiver operating characteristic curve (AUC). The classifiers were implemented in scikit-learn (Pedregosa et al., 2011) v0.23.2. Feature standardization was done by referring to the mean and the standard deviation of the training samples. Between condition comparisons were conducted by paired-sample *t*-test and repeated measures analysis of variance (ANOVA) in SPSS (v23). When the assumption of sphericity was violated (Mauchly’s Test of Sphericity), the degrees of freedom were adjusted using the Huynh-Feldt correction (Abdi, 2010).

## 3. Results

### 3.1. Behavioral data

**FIGURE 3:**
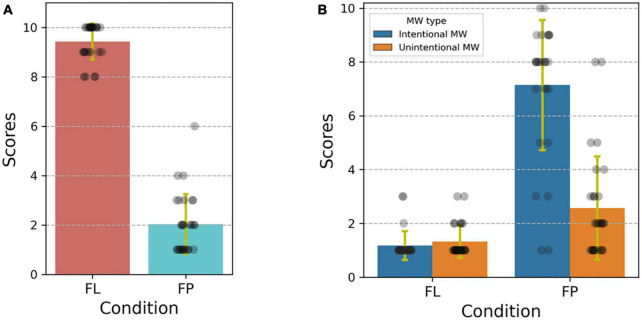
**(A)** Error bar and scatter plot of participant rating scores for percent of time focused on the video under the focused learning condition (FL) and future planning condition (FP). 1: < 10%, 2:10% to 20%, 3: 20% to 30%, etc. Each dot is a score from one video, error bar represents plus and minus one standard deviation. **(B)** Error bar and scatter plot of rating scores for percent of time in intentional and unintentional mind wandering. Score meanings are the same as above.

Figure 3A shows rating scores (collected after each video) corresponding to percent of time focused on the video (1 corresponding to <10%, 10 corresponding to 90–100%, see 2.3). The average values were 9.43 ± 0.73, (focused learning condition) and 2.04 ± 1.21, (future planning condition). Paired-sample *t*-tests showed significantly higher [*t*(27) = 29.752, *p* < 0.001] scores in the focused learning condition.

Figure 3B gives the intentional and unintentional mind wandering frequency ratings under each condition. Two-way, repeated measures ANOVA with condition type and mind wandering type (intentional vs. unintentional) as within-participant factors revealed a significant main effect of wandering type [*F*(1,27) = 43.872, *p* < 0.001] and condition type [*F*(1,27) = 268.927, *p* < 0.001]; significantly higher mind wandering rates were reported in the future planning condition. The interaction effect was also significant [*F*(1,27) = 40.673, *p* < 0.001]. Bonferroni-adjusted comparisons indicated that, participants reported significantly higher intentional mind wandering rates in the future planning condition compared to the focused learning condition (*p* < 0.001, 95% CI of the difference = 5.183 to 6.817), and they also reported significantly higher unintentional mind wandering rates in the future planning condition compared to the focused learning condition (*p* = 0.001, 95% CI of the difference = 0.722 to 2.492). Moreover, under the future planning condition, intentional mind wandering rates were significantly higher than unintentional mind wandering rates (*p* < 0.001, 95% CI of the difference = 2.933 to 5.567), while there was no significant difference between intentional and unintentional mind wandering in the focused learning condition. These trends accord with prior work and support the validity of our experimental paradigm.

### 3.2. Classification

#### 3.2.1. Within participant classification

**FIGURE 4:**
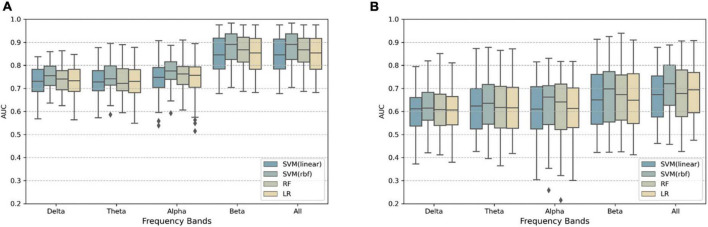
**(A)** Boxplots showing the AUC scores of within-participant classification based on different classifiers and frequency bands. An individual data point represents the result of one fold of cross-validation, so the total number of data points for each frequency band and each classifier was 14(participants) × 5(folds) = 70. **(B)** Same as above but for cross-lecture prediction AUC. The total number of data points for each frequency band and each classifier was 14(participants) × 4(train-test video combinations) = 56.

Figure 4A shows AUC of within participant classification, comparing frequency bands and classifiers. When using Riemannian features, SVM(rbf) classifier with the concatenation of all bands’ features had the highest performance AUC = 0.876± 0.070, the performance for each participant is provided in Supplementary Table 1. A repeated measures two-way ANOVA with frequency bands and classifiers as the within-participant factors revealed a significant main effect of frequency bands [*F*(1.603,110.577) = 130.929, *p* < 0.001] as well as classifiers [*F*(1.492,102.95) = 46.810, *p* < 0.001]. The interaction effect was not found to be significant [*F*(5.948,410.379) = 1.884, *p* = 0.083]. *Post-hoc* tests (Bonferroni-adjusted) indicated differences between SVM(rbf) and the other classifiers, LR, RF, and SVM(linear), were significant (*p* < 0.001), and differences between all pairwise combinations of the five bands (*p* < 0.05) were significant, except for all vs. beta band, delta vs. theta band.

#### 3.2.2. Cross-lecture classification

Figure 4B shows AUC of cross-lecture classification, comparing frequency bands and classifiers. The SVM(rbf) classifier with the concatenation of all bands’ features again had the highest performance AUC = 0.703± 0.108, performance for each participant is provided in Supplementary Table 2. Half of participants had higher average (across combinations) AUC than 0.7. Two-way, repeated measures ANOVA indicated that the main effect of classifier was significant [*F*(1.260,69.288) = 8.425, *p* = 0.003]; the main effect of frequency bands was also significant [*F*(1.979,108.857) = 12.564, *p* < 0.001]. There was no significant interaction effect found [*F*(4.154,228.489) = 0.890, *p* = 0.474]. *Post-hoc* tests (Bonferroni-adjusted) indicated differences between SVM(rbf) and the other classifiers were significant (*p* < 0.001); in terms of frequency bands, the combination of the four bands had significantly higher AUC than the delta, theta, and alpha bands alone (*p* < 0.05).

#### 3.2.3. Training sample size effects for cross-lecture classification

**FIGURE 5:**
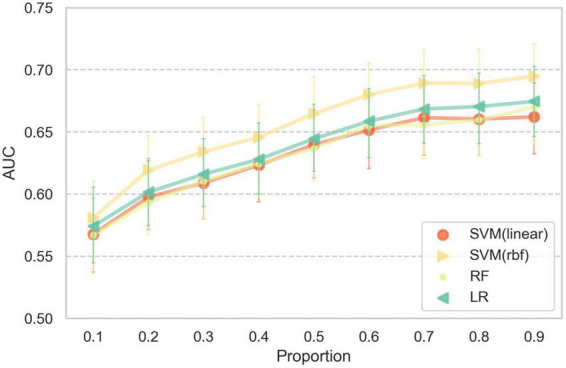
Error bar plot of AUC scores for different proportions of the training set. Error bars indicate plus and minus one standard deviation across participants.

The AUC of cross-lecture prediction for different proportions of training samples is shown in Figure 5. The AUC of all four classifiers increased as the training sample size increased, with SVM(rbf) performing the best. When 70% of the training samples were used, the average cross-lecture prediction AUC was close to that based on the full training set (mean AUC 0.689).

## 4. Discussion

We used Riemannian geometry based features and several machine learning methods to discriminate focused learning from mind wandering during video lecture viewing. The SVM(rbf) classifier performed best among the classifiers, and by combining Riemannian-based features from delta, theta, alpha, and beta bands, the classifier could detect mind wandering at 0.876 AUC for within participant prediction and 0.703 AUC for within participant cross-lecture prediction, on average.

The primary contribution of our study lies in the use of Riemannian geometry for feature extraction, which resulted in higher accuracy in detecting mind wandering states. Furthermore, we were able to achieve this performance using data collected by a limited-channel EEG system that is deployable in realistic conditions. Our findings also indicate that our methods for collecting training data sets in a short time period suitable for future online use, while carefully controlling conditions during data collection, can offer a viable solution for real-time decoding of mind wandering states.

Regarding online learning (or similar) scenarios, Conrad and Newman used an auditory odd-ball stimuli while participants watched lecture videos (Conrad and Newman, 2021), but decoding was not done in that work. Dhindsa et al. (2019) conducted an experiment in a real classroom setting, used common spatial patterns to extract features, and found slightly higher than random-chance level average F1 scores for mind wandering state classification, partly due to limited and imbalanced training sets. Our paradigm of focused learning versus future planning conditions was designed to collect non-mind-wandering and mind wandering data while avoiding interruption of the ongoing task process, which makes our results more generalizable to real video-based learning scenarios. Additional advantages include more balanced sample sizes between mind wandering and non-wandering states (Figure 2A) and better control of the order of conditions. Compared to Dhindsa et al. (2019) decoding performance was high under our realistic video-based distance learning scenario. Figure 5 shows that reliable prediction could be made with as short as 9 min of EEG recording for parameter fitting, allowing a system based on our method to be practically deployed to help learners or supervisors improve the efficacy of learning. In addition, our methods are also useful for investigating the role of mind wandering in the learning process, since our methods realize near-real-time detection (2-s update rate) of the occurrence of mind wandering.

We separately tested decoding performance using different frequency bands, which may subserve different functional roles during mind wandering (Kam et al., 2022), and we observed that beta band gave the highest prediction performance among the 4 frequency bands in both classification pipelines. Kaushik et al. (2022) reported theta and alpha gave the highest performance. Dhindsa et al. (2019) compared alpha, theta, and beta band (beta1:13–18 Hz, and beta2: 19–30 Hz), and their results showed that beta band (both beta1 and beta2) had the highest performance in a portion of the participants. From these disparate findings, the relative contribution of beta band activity may vary across individuals and experimental tasks.

Compared to former studies that used a single lecture video and ignored individual differences (Conrad and Newman, 2019, 2021), for the focused learning condition, we ensured high engagement through lecture content related discussion, facial video recording, expectation of post-lecture questions, and choice of self-selected learning materials of interest. For the future planning condition, cue images provided by the participants were highly personally relevant and thus better able to provide an environment conducive to mind wandering thoughts. Figure 3A shows that our methods for increasing motivation to pay attention during the focused learning condition were fairly successful, though the occurrence of mind wandering is inevitable during task (Seli et al., 2016b), the vast majority of participants reported that less than 10% of total focused learning condition duration was spent on mind wandering thoughts. Interestingly, the proportions of the two kinds of mind wandering (intentional and unintentional) under both conditions was similar to numbers from Seli et al. (2016a), with our focused learning condition corresponding to their difficult task and our future planning condition corresponding to their easy task, though there are differences in the tasks (Seli et al., 2015).

In the future planning condition, we took measures to increase the likelihood that the participants self-generate thoughts unrelated to the lectures. Any future planning thoughts generated by following the instructions (cue-initiated) do not conform to the traditional definition of mind wandering thoughts, which are self-initiated (Smallwood and Schooler, 2015). However, we expect participants to also generate self-initiated mind wandering thoughts under this experimental environment because they are watching videos which are very uninteresting and have no need to answer questions afterward. We confirmed the presence of self-initiated mind wandering via rating scales at the end of the videos. Our paradigm is similar to that of Zhigalov et al. (2019), which instructed future planning in contrast to mindfulness meditation. We chose future planning instructions to build an environment conducive to mind wandering, because mind wandering predominantly involves self-relevant and goal-directed planning (Baird et al., 2011). Seli et al. (2017) also found that the content of both unintentional and intentional mind wandering tend to be future-oriented.

We used the ASR method mainly to correct large-amplitude artifacts, and since this method is not perfect, some residual effects of noise can leak through this step. Some research have reported that eye-related information is useful for mind wandering detection (Bixler et al., 2015; Chen et al., 2022). Since our main purpose is accurate detection and a practical system, not necessarily one solely limited to signals of neural origin, we did not pursue perfect noise removal. Though eye-movement artifacts may have played a role, covariance-based features are known to be less sensitive to noise (Aydarkhanov et al., 2020).

There are several limitations to the current study. First, in our rating scales, many participants reported less than 10% of time spent in intentional and unintentional mind wandering during the focused learning condition, but participants may have been able to give more detailed information had we used a continuous rating scale instead of a 10-point scale. We asked participants to report the percentage of time spent in intentional and unintentional wandering after each video, but the accuracy of this estimate may need to be verified with the aid of key press during the experiment, i.e., the key press protocol currently used does not distinguish between intentional and unintentional wandering. Second, it is possible that the similarity in video content related neural activity underpinned the classification, instead of activity related to mind wandering. We have analyzed the selection of the interesting and uninteresting videos by participants, and found that, for interesting videos, 2 videos were repeatedly selected (the most-commonly selected video was selected 4 times). For uninteresting videos, 1 video was repeatedly selected (2 times). Future studies should verify our results on larger samples and with different lecture contents. Third, our study only conducted within individual classification, as a step toward the more generalizable inter-individual classification, which we plan in future work.

An ideal pBCI system for daily usage should be usable at any moment. However, noise from hardware, variability of brain activity, and inter-individual variability make this very difficult to achieve. A solution is reflected in the work of Aricò et al. (2016), in which a short calibration period was used to collect task-related signals. The result of the current 8-channel study shows that even when the training data duration is limited to 9 min, our methods could still obtain high cross-lecture prediction accuracy. The approach combining a limited-channel EEG system with a short calibration period shows promise for future research.

## Data availability statement

The raw data supporting the conclusions of this article will be made available by the authors, without undue reservation. Pre-processed data and code are available at https://osf.io/7mj5e/.

## Ethics statement

The study involving human participants was reviewed and approved by the Ethics Review Committee of the School of Psychology of Beijing Normal University. Written informed consent to participate in this study was provided by the participants or their legal guardian/next of kin.

## Author contributions

ST and ZL conceptualized the study and wrote the manuscript. ST and YL collected and analyzed the data. All authors approved the submitted version.

## Footnotes

1
https://www.icourse163.org/
