# Supplementary material for: Mind wandering state detection during video-based learning via EEG
Source: Front Hum Neurosci. 2023 May 30;17:1182319. doi: 10.3389/fnhum.2023.1182319 (PMC10267732; doi:10.3389/fnhum.2023.1182319)
Supplement: Supplementary file 1 [file Table_1.DOCX]

**Supplementary Table 1.** AUC scores (mean$\pm$std) for within participant prediction. The features were the concatenation of the 4 frequency bands (delta, theta, alpha, and beta). LR: logistic regression. RF: random forest. SVM(linear): support vector machine with linear kernel. SVM(rbf): support vector machine with radial basis function kernel.

| **Participant** | LR | RF | SVM(linear) | SVM(rbf) |
| --- | --- | --- | --- | --- |
| P1 | 0.93$\pm$ 0.02 | 0.94$\pm$ 0.02 | 0.91$\pm$ 0.03 | 0.94$\pm$ 0.02 |
| P2 | 0.86$\pm$ 0.01 | 0.81$\pm$ 0.03 | 0.79$\pm$ 0.02 | 0.82$\pm$ 0.04 |
| P3 | 0.92$\pm$ 0.03 | 0.84$\pm$ 0.05 | 0.82$\pm$ 0.03 | 0.85$\pm$ 0.04 |
| P4 | 0.73$\pm$ 0.04 | 0.74$\pm$ 0.04 | 0.73$\pm$ 0.03 | 0.76$\pm$ 0.03 |
| P5 | 0.8$\pm$ 0.04 | 0.89$\pm$ 0.02 | 0.9$\pm$ 0.03 | 0.91$\pm$ 0.03 |
| P6 | 0.91$\pm$ 0.02 | 0.84$\pm$ 0.02 | 0.8$\pm$ 0.03 | 0.85$\pm$ 0.03 |
| P7 | 0.79$\pm$ 0.03 | 0.95$\pm$ 0.01 | 0.95$\pm$ 0.02 | 0.96$\pm$ 0.00 |
| P8 | 0.82$\pm$ 0.05 | 0.89$\pm$ 0.03 | 0.92$\pm$ 0.02 | 0.93$\pm$ 0.03 |
| P9 | 0.72$\pm$ 0.03 | 0.82$\pm$ 0.03 | 0.78$\pm$ 0.04 | 0.89$\pm$ 0.01 |
| P10 | 0.91$\pm$ 0.03 | 0.88$\pm$ 0.02 | 0.85$\pm$ 0.02 | 0.91$\pm$ 0.03 |
| P11 | 0.81$\pm$ 0.01 | 0.95$\pm$ 0.01 | 0.92$\pm$ 0.03 | 0.96$\pm$ 0.02 |
| P12 | 0.95$\pm$ 0.02 | 0.72$\pm$ 0.02 | 0.73$\pm$ 0.04 | 0.74$\pm$ 0.03 |
| P13 | 0.92$\pm$ 0.01 | 0.82$\pm$ 0.03 | 0.81$\pm$ 0.03 | 0.83$\pm$ 0.04 |
| P14 | 0.78$\pm$ 0.02 | 0.91$\pm$ 0.02 | 0.92$\pm$ 0.01 | 0.92$\pm$ 0.02 |
| Average | 0.85$\pm$ 0.07 | 0.86$\pm$ 0.07 | 0.85$\pm$ 0.08 | 0.88$\pm$ 0.07 |

**Supplementary Table 2.** AUC scores (mean$\pm$std) for cross-lecture prediction. The features were the concatenation of the 4 frequency bands (delta, theta, alpha, and beta).

| **Participant** | LR | RF | SVM(linear) | SVM(rbf) |
| --- | --- | --- | --- | --- |
| P1 | 0.7$\pm$ 0.07 | 0.73$\pm$ 0.09 | 0.67$\pm$ 0.08 | 0.75$\pm$ 0.08 |
| P2 | 0.64$\pm$ 0.09 | 0.62± 0.14 | 0.63$\pm$ 0.08 | 0.63$\pm$ 0.01 |
| P3 | 0.58$\pm$ 0.08 | 0.72± 0.07 | 0.57$\pm$ 0.06 | 0.69$\pm$ 0.09 |
| P4 | 0.56$\pm$ 0.07 | 0.55± 0.12 | 0.56$\pm$ 0.07 | 0.58$\pm$ 0.1 |
| P5 | 0.84$\pm$ 0.01 | 0.79± 0.06 | 0.82$\pm$ 0.02 | 0.79$\pm$ 0.04 |
| P6 | 0.71$\pm$ 0.07 | 0.69± 0.09 | 0.69$\pm$ 0.07 | 0.73$\pm$ 0.09 |
| P7 | 0.66$\pm$ 0.12 | 0.59± 0.05 | 0.65$\pm$ 0.12 | 0.61$\pm$ 0.12 |
| P8 | 0.77$\pm$ 0.02 | 0.74± 0.08 | 0.75$\pm$ 0.04 | 0.79$\pm$ 0.04 |
| P9 | 0.63$\pm$ 0.08 | 0.55± 0.04 | 0.61$\pm$ 0.09 | 0.61$\pm$ 0.06 |
| P10 | 0.72$\pm$ 0.05 | 0.58± 0.08 | 0.73$\pm$ 0.06 | 0.68$\pm$ 0.06 |
| P11 | 0.77$\pm$ 0.07 | 0.82± 0.03 | 0.76$\pm$ 0.09 | 0.81$\pm$ 0.01 |
| P12 | 0.51$\pm$ 0.03 | 0.55± 0.02 | 0.5$\pm$ 0.03 | 0.57$\pm$ 0.05 |
| P13 | 0.67$\pm$ 0.01 | 0.75± 0.11 | 0.66$\pm$ 0.04 | 0.76$\pm$ 0.07 |
| P14 | 0.82$\pm$ 0.06 | 0.81± 0.07 | 0.79$\pm$ 0.06 | 0.84$\pm$ 0.07 |
| Average | 0.68$\pm$ 0.11 | 0.68$\pm$ 0.12 | 0.67$\pm$ 0.11 | 0.70$\pm$ 0.11 |
